# Bacteria and Sewage

**Published:** 1900-05-05

**Authors:** 


### BACTERIA AND SEWAGE.

There are few matters concerning which it more behoves investors to be upon their guard than those which may possibly involve attempts at the premature commercial application of scientific discoveries; and we are much disposed to think that a word of caution may not be untimely with regard to the schemes of various projectors touching what is beginning to be called the bacteriological treatment of sewage. It has long been known that the ultimate results of spontaneous decomposition of the waste material poured forth from our great towns and cities are to resolve that material into harmless elementary substances, or into simple compounds which admit of various forms and methods of utilisation; and many endeavours were made, some five and twenty years ago, so to accelerate this decomposition as at once to remove nuisances from inhabited districts, and to convert them into forms which could be dealt with in a way to yield pecuniary profit. At that time the spontaneous decomposition in question was regarded as a purely "chemical" change, the dependence of which upon living organisms was not even suspected; and hence its acceleration or modification was aimed at by chemical additions or processes. A variety of methods of dealing with sewage were suggested by different persons, and many of them were of such a nature that, when tested upon small quantities in a laboratory, they appeared to justify expectations of decided pecuniary success—success which was to be derived, in most instances, from the manurial value of the products. Even then, as on so many other occasions, Wisdom cried aloud in the streets, and no man regarded her. The late General Scott, who had given much attention to the subject, and whose attainments as a chemist were second only to his skill as an engineer, declared on all occasions that it would cost twenty-five shillings to separate a pound's worth of fertilising material from the water and inert matter which composed the bulk of sewage. He warned local authorities that they must pay to be clean, and that the only practical question for them to decide was that of the cheapest method of accomplishing the desired result. Other companies were formed, however, the directors of which were sufficiently confident in the remunerative character of their respective patents and processes to offer to pay local authorities for their sewage, instead of asking to be paid to remove it; and these naturally carried the day. The aggregate of their share capital amounted in round numbers to about two millions sterling, and we believe that a large proportion of this has been wholly lost.

The modern discovery that the spontaneous decomposition of sewage is not a purely chemical process, but that it is initiated by, and dependent upon, the presence and multiplication of countless millions of bacteria, some of them aerobic and others anaerobic, has quite naturally suggested that the best method of dealing with one of the chief problems of town life may be to place the sewage under conditions eminently favourable to bacterial development in both the above-named forms. Different methods of accomplishing this object have been suggested, and have been made the subjects of experiment upon scales of different magnitude, and over longer and shorter periods of time. Many of the projectors are hopeful, a few are confident, some may perhaps be described as jubilant. Endeavours are being made, in more than one direction, to obtain the capital required for doing on a commercial basis what has been done successfully on an experimental one; and many mayors, town councillors, and other local authorities have been "approached" with seductive promises. The rates are to be reduced, nuisances will cease to exist, and pellucid rivers will swarm with savoury fish. We remember every word of this as an old, old story, which has not hitherto been justified by events. Bacteria have existed, and have worked their will upon animal refuse, for countless centuries; but they have never yet accomplished their scavenging with sufficient celerity to prevent this refuse from becoming a source of discomfort and of danger to large aggregations of men. To what extent the favourable conditions afforded by coke-filter beds and by sandy soil may add to their efficiency it is as yet too soon to declare; and a highly important economic question turns upon the length of time during which such filter beds and such soil will maintain the conditions which they are designed to secure. We do not deny the possibility of effectively promoting bacterial activity, nor dispute that such activity may yet be utilised for the removal of the chief difficulty by which urban sanitary authorities are beset. But we wish to remind all who are concerned of the history of many sewage undertakings in the past, and to point out that this history calls emphatically for caution in the present.

